# The Effect of COVID‐19 on Platelet Counts in Persistent and Chronic Adult ITP Patients: A Real‐World Study in China

**DOI:** 10.1002/jha2.70025

**Published:** 2025-03-29

**Authors:** Yujiao Zhang, Lei Yang, Zhongping Xu, Xin Zhou

**Affiliations:** ^1^ Department of Hematology The Affiliated Wuxi People's Hospital of Nanjing Medical University, Wuxi People's Hospital, Wuxi Medical Center, Nanjing Medical University Wuxi Jiangsu China; ^2^ Department of Critical Care Medicine The Affiliated Wuxi People's Hospital of Nanjing Medical University, Wuxi People's Hospital, Wuxi Medical Center, Nanjing Medical University Wuxi Jiangsu China

**Keywords:** COVID‐19, immune thrombocytopenia, thrombocytosis, TPO‐RA

## Abstract

**Objective:**

While coronavirus disease 2019 (COVID‐19)‐associated thrombocytopenia is well‐documented, its effects on immune thrombocytopenia (ITP) patients remain unclear. This study aimed to investigate the impact of COVID‐19 infection on platelet (PLT) dynamics in chronic ITP patients.

**Methods:**

This retrospective study analyzed 21 persistent and chronic ITP patients before and after mild COVID‐19 infection during China's December 2022 reopening, comparing platelet parameter changes with a focus on clinical characteristics of thrombopoietin receptor agonist (TPO‐RA) treated patients.

**Results:**

TPO‐RA treated patients demonstrated transient platelet surges peaking at 1 week postinfection, returning to baseline within 2–3 weeks, contrasting sharply with thrombocytopenia patterns in non‐ITP populations. This suggests synergistic effects between virus‐induced inflammatory cytokines and TPO‐RA may drive transient megakaryopoiesis.

**Conclusion:**

These findings underscore infection‐related PLT fluctuations in ITP, necessitating monitoring for thrombotic and bleeding risks and TPO‐RA dose optimization during infections.

**Trial Registration:**

The authors have confirmed clinical trial registration is not needed for this submission

## 1 Introduction

Immune thrombocytopenia (ITP) is an acquired autoimmune bleeding disorder characterized by an isolated decrease in peripheral blood platelet (PLT) count (usually less than 100 × 10^9^/L) without a definite causation. Recently, several investigations have revealed that complicated immune intolerance to their own PLTs might play a key role in PLT overdestruction or insufficient PLT production in ITP patients [1, 2].

Since the emergence of coronavirus disease 2019 (COVID‐19) as a serious public health concern worldwide, lots of COVID‐19 patients present hematological changes, in which thrombocytopenia, the most common, is reported up to 36% [3]. For COVID‐19 patients who are previously healthy, thrombocytopenia tends to be more commonly observed in severe cases, with a mild degree of thrombocytopenia. Severe thrombocytopenia, or even in cases of COVID‐19 with PLT counts of almost zero, is very rare; as reported, immune‐induced thrombocytopenia is far more likely to occur than other etiologies [4]. To date, there have been few reports about the effect of COVID‐19 on patients with ITP, who are at high risk of bleeding and thrombocytosis, especially those who have previously used thrombopoietin receptor agonists (TPO‐RA) [5, 6, 7]. In December 2022, with the full liberalization of COVID‐19 epidemic control in China, we retrospectively analyzed the changes in PLT counts, adverse reactions, and effects on subsequent treatment after COVID‐19 infection in patients with previously diagnosed ITP, particularly persistent and chronic ITP in our department.

## 2 Materials and Methods

In this retrospective study, we investigated 21 patients with ITP and COVID‐19 from the Affiliated Wuxi People's Hospital of Nanjing Medical University by telephone follow‐up, which included persistent and chronic adult ITP patients with a confirmed diagnosis of COVID‐19 from December 1, 2022 to February 1, 2023. All patients expressed informed consent to participate in the study by telephone.

The criteria of patients for follow‐up were aged ≥ 18 years with persistent and chronic ITP, confirmation of COVID‐19 infection within 48 h of onset of COVID‐19 infection by a COVID‐19 antigen test kit on nasopharyngeal swabs or by polymerase chain reaction (PCR) on nasal and throat swab samples or serology.

The efficacy criteria for TPO‐RA were: complete response (CR) means PLT counts ≥ 100 × 10^9^/L with no bleeding symptoms after treatment; response (R) means PLT counts 30–100 × 10^9^/L and at least twice higher than baseline PLT counts with no bleeding symptoms after treatment; no response (NR) means PLT counts < 30 × 10^9^/L or less than twice the minimum PLT level or bleeding.

Throughout the period from the patient's diagnosis of COVID‐19 to cure, we collected all the patient's data, including age, sex, type of ITP, ongoing treatment regimen, vaccination status, severity of COVID‐19, adverse reactions, baseline PLT count before COVID‐19 infection, and absolute PLT counts 1–3 weeks after COVID‐19 infection. The baseline PLT counts were defined as the median value of ≥ 3 PLT measurements obtained during the month preceding COVID‐19 diagnosis, while postinfection PLT counts were designated as values recorded within 24 h following virological confirmation of COVID‐19 infection.

Data were summarized descriptively using Excel (Microsoft) software. Continuous variables with normal and non‐normal distributions were presented as medians and ranges, with statistical values retained to two decimal places. The Mann–Whitney *U* test was employed to compare differences between groups for non‐normally distributed data. All analyses were conducted using SPSS 26.0 for Windows (SPSS), with statistical significance defined as a *p* < 0.05.

## 3 Results

A total of 21 (9 males and 12 females) ITP patients who were diagnosed with COVID‐19 were followed up. The median age was 48 (18, 68) years. Among them, 15 (71.43%) were chronic ITP, and 6 (28.57%) were persistent ITP. In addition, there were no newly diagnosed ITP or recurrent cases after COVID‐19 infection in our investigation. Before COVID‐19 infection, 13 (61.90%) patients were treated with TPO‐RA, including 9 (42.86%) with eltrombopag (EPAG) and 4 (19.05%) with hetrombopag (HPAG). Besides, two (9.52%) patients were receiving treatment with methylprednisolone (mPSL), whereas the remaining six patients (28.57%) did not receive any treatment due to self‐discontinuation of medication, asymptomatic or poor response to previous treatment, and were in the regular follow‐up stage. Furthermore, eight patients (38.09%) had received at least two doses of the anti‐COVID‐19 vaccine. The demographic and clinical characteristics are shown in Table 1.

**TABLE 1:** Clinical and demographic features of chronic and persistent ITP with COVID‐19 infection.

Patient, no	Age, years	Sex	Type of ITP	ITP treatment before COVID‐19	ITP treatment after COVID‐19	COVID‐19 vaccination	Anticoagulant therapy	Thrombosis	COVID‐19 severity
1	45	M	C	EPAG 75 mg	None	No	No	No	Mild
2	69	F	C	EPAG 75 mg	EPAG 50 mg	No	No	No	Mild
3	67	F	C	EPAG 75 mg	EPAG 50 mg	No	No	No	Mild
4	29	F	C	HPAG 2.5 mg	None	No	No	No	Mild
5	46	F	C	None	None	No	No	No	Mild
6	49	M	C	None	None	No	No	No	Mild
7	40	M	C	mPSL 8 mg	mPSL 6 mg	No	No	No	Mild
8	52	F	C	None	None	No	No	No	Mild
9	68	F	P	HPAG 5.0 mg	HPAG 2.5 mg	Yes (three)	No	No	Mild
10	66	M	C	None	None	Yes (three)	No	No	Mild
11	53	F	C	None	IVIg; TPO; PLT transfusions	No	No	No	Mild
12	19	M	C	None	None	No	No	No	Mild
13	31	M	P	mPSL 8 mg	None	Yes (three)	No	No	Mild
14	46	F	P	HPAG 2.5 mg	None	Yes (three)	No	No	Mild
15	50	F	P	HPAG 2.5 mg	HPAG 5.0 mg	Yes (three)	No	No	Mild
16	46	M	C	EPAG 25 mg	None	No	No	No	Mild
17	67	F	C	EPAG 25 mg	PLT transfusions	No	No	No	Moderate
18	51	M	C	EPAG 50 mg	None	Yes (two)	No	No	Mild
19	44	F	P	EPAG 50 mg	None	Yes (three)	No	No	Mild
20	51	F	C	EPAG 25 mg	None	No	No	No	Mild
21	39	M	P	EPAG 50 mg	APAG 20 mg	Yes (three)	No	No	Mild

*Note*: The severity of COVID‐19 infection was determined as follows: “mild” indicates asymptomatic or not requiring oxygen therapy; “moderate” requires oxygen therapy on hospitalization; and “severe” requires ventilator management or intensive care unit (ICU) management.

Abbreviations: APAG, avatrombopag; C, chronic; EPAG, eltrombopag; F, female; HPAG, hetrombopag; IVIg, intravenous immunoglobulin; M, male; mPSL, methylprednisolone; P, persistent; PLT, platelet; TPO, thrombopoietin.

**FIGURE 1:**
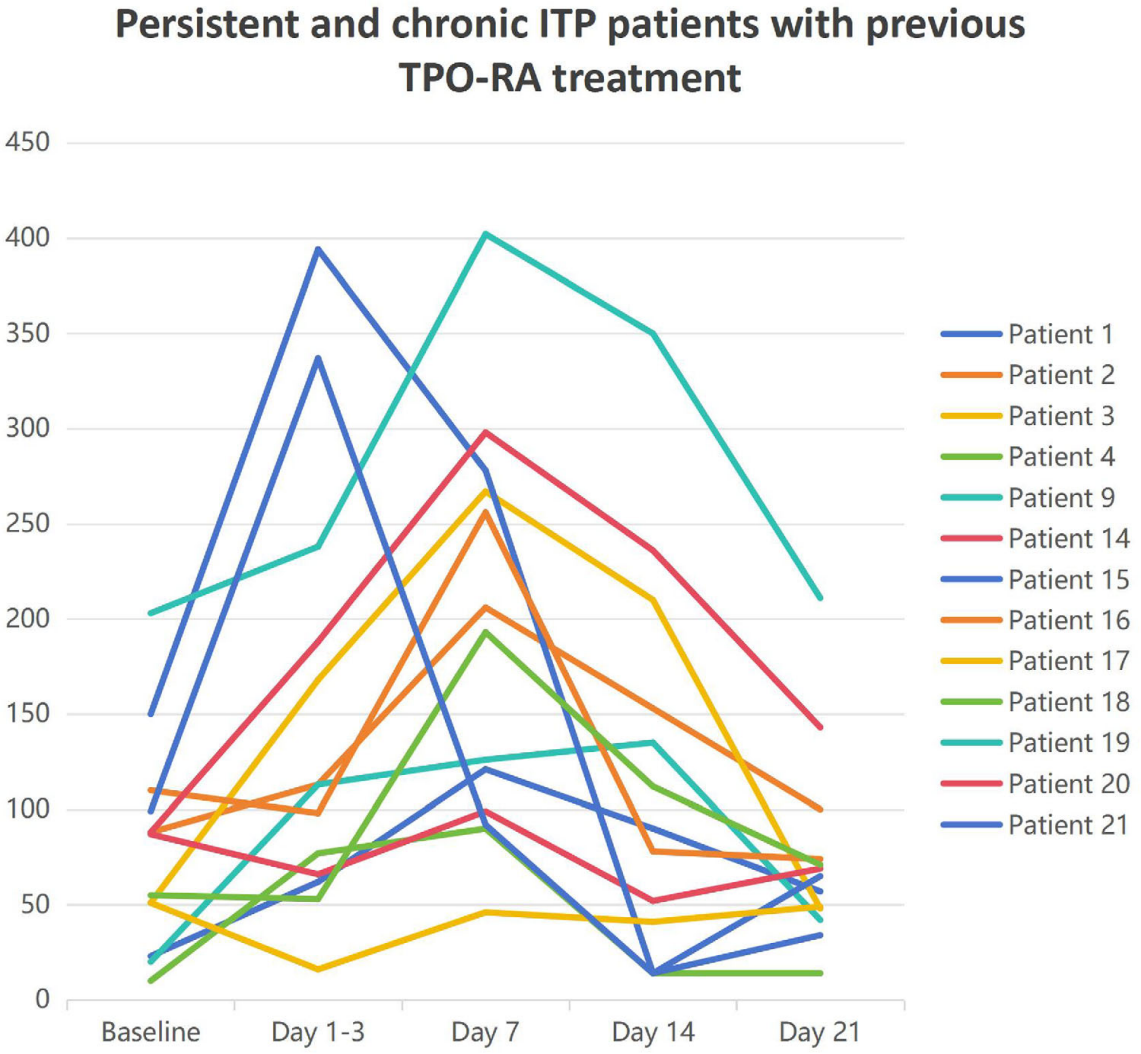
Temporal variations in PLT counts among adult persistent and chronic ITP patients with prior TPO‐RA therapy: Comparative analysis at baseline (pre‐COVID‐19 infection) and postinfection time points (Days 1–3, 7, 14, 21 following symptoms appear).

Following COVID‐19 infection, patients treated with TPO‐RA exhibited transient thrombocytosis. The median baseline PLT count was 88 × 10^9^/L, peaking at 168 × 10^9^/L 1 week postinfection and returning to baseline within 2–3 weeks, as shown in Table 2 and Figure 1. Notably, 69.23% (9/13) of TPO‐RA treated patients demonstrated a > 2.5‐fold PLT increase, exemplified by Patient 3 (51→267 × 10^9^/L) and Patient 19 (203→402 × 10^9^/L), with four cases requiring dose reduction due to PLT counts exceeding 300 × 10⁹/L to mitigate thrombotic risks. In contrast, corticosteroid‐treated and untreated patients displayed milder PLT elevations, such as Patient 7 (40→78 × 10⁹/L). Statistical analysis revealed that the TPO‐RA group had a significantly greater median PLT increase (+168 × 10⁹/L) at 1 week postinfection compared to the non‐TPO‐RA group (corticosteroid‐treated and untreated patients: median +46 × 10⁹/L; Mann–Whitney *U* test = 6.5, *p *= 0.002), with a large effect size (*r* = 0.63). No thromboembolic or hemorrhagic events were observed in our patients.

**TABLE 2:** Longitudinal platelet counts data.

	Baseline, ×10^9^/L	Days 1–3, ×10^9^/L	Day 7, ×10^9^/L	Day 14, ×10^9^/L	Day 21, ×10^9^/L
Patient 1	23	62	121	90	57
Patient 2	88	113	206	153	100
Patient 3	51	168	267	210	48
Patient 4	10	77	90	14	14
Patient 9	20	113	126	135	42
Patient 14	87	66	99	52	69
Patient 15	150	394	278	14	65
Patient 16	110	98	256	78	74
Patient 17	51	16	46	41	49
Patient 18	55	53	193	112	71
Patient 19	203	238	402	350	211
Patient 20	88	188	298	236	143
Patient 21	99	337	92	14	34

In our study, only one patient (patient 17) developed a moderate COVID‐19 infection and was hospitalized for severe pneumonia therapy. After infection, petechiae appeared all over the skin, and the PLT count decreased from baseline 51 × 10^9^/L to 16 × 10^9^/L in this patient. Then, she was immediately treated with PLT transfusion in the emergency and hospitalized later. After 1 week (Day 7) of being diagnosed with COVID‐19, the patient's PLT count rebounded to 46 × 10^9^/L. On Day 14, the patient was discharged from the hospital without any fever or petechiae, and the test for COVID‐19 was negative as well as the PLT count, which reached 41 × 10^9^/L. Fortunately, this patient did not develop severely fatal bleeding symptoms during the COVID‐19 infection, and no thrombocytosis was discovered.

## 4 Discussion

Thrombocytopenia has been reported to be the most common hematological change in COVID‐19 patients, usually presenting with mild forms, and is often observed in cases with severe COVID‐19 [3]. However, severe thrombocytopenia or even almost zero with PLT counts are very rare. Mohammad et al. reported a case of absolute thrombocytopenia (PLT count of 2 × 10^9^/L) after COVID‐19 infection, and eventually, they thought that COVID‐19‐triggered immune‐induced thrombocytopenia was probably the best explanation for their patients [4]. Emerging evidence suggests that thrombocytopenia in COVID‐19 patients is significantly associated with elevated in‐hospital mortality and adverse clinical outcomes. Importantly, the resolution of thrombocytopenia has been observed to correlate with subsequent clinical improvement, indicating its potential value as a dynamic prognostic indicator in disease progression [8].

Noteworthy, there are a few investigations that have reported the exact different findings in ITP patients with COVID‐19 infection from newly diagnosed COVID‐19‐related ITP. So far, three teams have found that chronic ITP patients had a transient increase in PLT counts after COVID‐19 infection. de la Cruz‐Benito et al. were the first team to put forward this finding. They had a diverse range of previous treatment regimens in their case series, but they only showed that chronic ITP patients had increased PLT counts after COVID‐19 and needed treatment adjustment, as well as did not compare the magnitude of PLT counts increase under different treatments [5]. The case series by Pantic et al. of chronic ITP patients who were treated with TPO‐RA sustained remission for more than 1 month clearly indicated that the increased PLT counts after COVID‐19 were associated with the treatment of TPO‐RA, and the peak PLT counts occurred on Day 7 after infection. Instead, the occurrence of events in their patients, such as pulmonary embolism and fatal intracranial hemorrhage, validates the importance and necessity of closely monitoring the PLT counts of chronic ITP patients and adjusting the treatment timely [6]. On the basis of the former two teams, Wang et al. expanded the sample size and presented more detailed conclusions in children with chronic ITP [7]. Furthermore, our study encompassed a substantial number of cases, including not only adult patients with prior TPO‐RA but also untreated individuals and those receiving glucocorticoid monotherapy, with additional representation from six persistent ITP cases.

However, Mingot et al. reported that 47% of ITP patients required therapeutic intervention for thrombocytopenia following COVID‐19 [9], while Lapietra et al. highlighted that not all COVID‐19‐infected ITP patients developed thrombocytosis [10]. These findings appear contradictory to our observations yet share underlying consistencies. The discrepancies likely stem from heterogeneity in treatment histories across study cohorts and variability in COVID‐19 severity. Notably, neither of these prior studies performed stratified outcome analyses based on preinfection ITP therapeutic regimens. Our data demonstrate that TPO‐RA‐treated patients constitute a distinct subgroup in whom COVID‐19 infection transiently reverses baseline thrombocytopenia. Mechanistically, TPO‐RA‐induced megakaryocyte (MK) priming may synergize with COVID‐19‐driven interleukin‐6 (IL‐6) surge, increasing PLT counts. Furthermore, virus‐mediated immunosuppression could reduce PLT auto‐antibody production, particularly in patients already benefiting from TPO‐RA's immunomodulatory effects on T‐regulatory cell homeostasis. On the other hand, the majority of ITP patients who developed thrombocytopenia after COVID‐19 infection in the above two studies were severely ill patients who required hospitalization, which is consistent with Patient 17, who was hospitalized with severe pneumonia and had severe thrombocytopenia during hospitalization and even required PLT transfusions. As a result, we found that only chronic and persistent ITP patients who had mild COVID‐19 infections and had previously used TPO‐RA had transient PLT elevation.

Based on current studies, it is known that in ITP patients, the possible mechanism for COVID‐19‐associated thrombocytosis can be analyzed from the aspects of decreased PLT destruction and increased PLT production. First, some research has shown that increased thrombopoietin (TPO) levels can be detected in the plasma of recovered patients with severe acute respiratory syndrome (SARS) patients in 2003, and the pathogens of COVID‐19 and SARS are both coronaviruses. Therefore, the study has suggested that TPO may be the cause of increased PLT counts in COVID‐19 patients [5, 11]. TPO can trigger megakaryocytes (MKs) to express Von Willebrand factor (vWF), which is upregulated during periods of stress such as inflammation due to COVID‐19 [12]; then increased vWF stimulates MKs to produce PLTs through glycoprotein (GP)Ib/vWF [13]. However, Wang et al. detected TPO levels of two ITP patients in TPO‐RA before and after COVID‐19 and did not find any increase in TPO levels [7], which may be due to the small number of samples, so this mechanism needs to be further validated. Moreover, the inflammatory mediator, IL‐6, can induce MKs to proliferate and differentiate into mature PLTs after COVID‐19 and also can enhance the viability of MKs, thereby increasing PLT counts [13]. Second, ITP patients with COVID‐19 can also have lymphopenia. After COVID‐19, both T lymphocytes and B lymphocytes decrease, resulting in a reduced sustained immune response, which reduces the production of PLT antibodies and increases the PLT counts [14].

Additionally, there are two ITP patients with single corticosteroids in our case series, and we have observed that they also experienced thrombocytosis during COVID‐19 infection, but to a lesser extent than patients with TPO‐RA. Therefore, we think that TPO‐RA plays a key role in thrombocytosis caused by ITP patients with COVID‐19 infection. It is reported that sensitizers can improve the initial response to TPO‐RA in ITP patients by increasing the levels of transforming growth factor‐β (TGF‐β) and tumor necrosis factor‐α (TNF‐α), and a significant increase in tissue biomarkers of TGF‐β can be detected in paraffin lung samples from COVID‐19 patients [7, 15]. The ITP patients in our study all responded to TPO‐RA, so we believe that the increase of TGF‐β can enhance the response of TPO‐RA to ITP patients.

According to the current global management guidelines, the risk of bleeding from COVID‐related ITP is high, and it may be life‐threatening at any time due to severe bleeding, so it is necessary to use corticosteroids in time to control bleeding symptoms. Moreover, corticosteroids and intravenous immunoglobulin (IVIG) are still the first‐line treatment options for COVID‐related ITP, but TPO‐RA is merely reserved for refractory ITP due to its risks, such as hepatotoxicity and thrombosis. As for persistent and chronic ITP patients with TPO‐RA in COVID‐19, in order to avoid bleeding and thrombosis, it is essential to closely monitor bleeding and coagulation indicators and adjust the treatment regimen in time.

Looking back at our investigation, the majority of patients had a significant increased PLT counts after COVID‐19 infection, but it is worthy to note that patients who respond to TPO‐RA are much more likely to have an increase in PLT count than those who respond to corticosteroid therapy, and have no regular treatment. Then, some patients need to adjust the dose of TPO‐RA or even anticoagulant therapy. With our timely intervention, we were fortunate to have no thrombocytosis or hemorrhagic events in our patients, and no deaths were observed during follow‐up.

Our study has several limitations that warrant consideration. Firstly, the retrospective single‐center design inherently constrained case ascertainment due to the potential underreporting of mild or asymptomatic COVID‐19 infections during the post‐restriction period, particularly among ITP patients avoiding medical visits, which may bias population‐level estimates of thrombocytosis prevalence. Secondly, while we adjusted for key clinical covariates, vaccination status (including vaccine type and booster doses) was not incorporated into the regression framework despite emerging evidence of vaccine‐mediated immunomodulation in ITP. Thirdly, the absence of longitudinal follow‐up beyond 3 months precludes definitive conclusions regarding whether transient platelet elevation alters long‐term relapse trajectories or modifies disease chronicity patterns.

## 5 Conclusion

Our study demonstrates that persistent and chronic ITP patients treated with TPO‐RA have a significantly transient increase in PLT counts about 1 week after mild COVID‐19 infection and fall back to baseline 2–3 weeks after infection. Further, the above phenomenon can also occur in patients with a single corticosteroid and untreated patients but to a lesser extent than patients with TPO‐RA.

## Author Contributions

L.Y. designed the research and revised the paper; Y.Z. and Z.X. performed the research and collected the data; L.Y. analyzed the data; X.Z. interpreted the data; Y.Z. wrote the paper.

## Ethics Statement

All patients expressed informed consent to participate in the study by telephone.

## Conflicts of Interest

The authors declare no conflicts of interest.

## Data Availability

All data created or analyzed during this study are included in this article. Further inquiries can be directed to the corresponding author.
